# Epidermal Growth Factor Receptor Transactivation Is Required for Mitogen-Activated Protein Kinase Activation by Muscarinic Acetylcholine Receptors in HaCaT Keratinocytes

**DOI:** 10.3390/ijms151121433

**Published:** 2014-11-21

**Authors:** Wymke Ockenga, Sina Kühne, Simone Bocksberger, Antje Banning, Ritva Tikkanen

**Affiliations:** Institute of Biochemistry, Medical Faculty, University of Giessen, Friedrichstrasse 24, D-35392 Giessen, Germany; E-Mails: Wymke.Ockenga@biochemie.med.uni-giessen.de (W.O.); Sina.Kuehne@biochemie.med.uni-giessen.de (S.K.); simonebocksberger@web.de (S.B.); Antje.Banning@biochemie.med.uni-giessen.de (A.B.)

**Keywords:** G protein coupled receptor, epidermal growth factor, acetylcholine, muscarinic receptor, transactivation, mitogen-activated protein kinase, keratinocytes, matrix metalloprotease

## Abstract

Non-neuronal acetylcholine plays a substantial role in the human skin by influencing adhesion, migration, proliferation and differentiation of keratinocytes. These processes are regulated by the Mitogen-Activated Protein (MAP) kinase cascade. Here we show that in HaCaT keratinocytes all five muscarinic receptor subtypes are expressed, but M_1_ and M_3_ are the subtypes involved in mitogenic signaling. Stimulation with the cholinergic agonist carbachol leads to activation of the MAP kinase extracellular signal regulated kinase, together with the protein kinase Akt. The activation is fully dependent on the transactivation of the epidermal growth factor receptor (EGFR), which even appears to be the sole pathway for the muscarinic receptors to facilitate MAP kinase activation in HaCaT cells. The transactivation pathway involves a triple-membrane-passing process, based on activation of matrix metalloproteases, and extracellular ligand release; whereas phosphatidylinositol 3-kinase, Src family kinases or protein kinase C do not appear to be involved in MAP kinase activation. Furthermore, phosphorylation, ubiquitination and endocytosis of the EGF receptor after cholinergic transactivation are different from that induced by a direct stimulation with EGF, suggesting that ligands other than EGF itself mediate the cholinergic transactivation.

## 1. Introduction

Acetylcholine (ACh) is mainly known as a neurotransmitter, but ACh and its receptors also act as mediators of cell communication in non-neuronal cells. The existence and importance of non-neuronal cholinergic systems has gained high attention and acceptance over the last two decades (reviewed in [1,2]). Two classes of ACh receptors mediate the effects of cholinergic stimulation. The nicotinic acetylcholine receptors (nAChR) are ion channels, whereas the muscarinic receptors (mAChR) belong to the family of G protein coupled receptors (GPCR) which are coupled to heterotrimeric guanine nucleotide binding proteins (G proteins). The mAChR family consists of five receptor subtypes referred to as M_1_, M_2_, M_3_, M_4_ and M_5_ receptors. Depending on the G protein type they are associated with, they activate different signal transduction pathways. M_1_, M_3_ and M_5_ preferentially couple to G proteins with an α_q/11_ subunit, whereas M_2_ and M_4_ receptors mainly couple to G proteins with the inhibitory α_i/o_ subtypes.

The human skin utilizes non-neuronal ACh signaling to regulate cell differentiation and tissue maintenance [3,4], and ACh appears to play a role in wound healing [5,6,7]. Various subunits of the nAChR and distinct mAChR subtypes are expressed, depending on the skin layer and the cell type [8,9,10], and high levels of ACh are detectable in the epidermis [11,12]. Furthermore, the cholinergic signals are important for proliferation, differentiation and migration of keratinocytes and other cell types within the layers of the skin [13,14,15,16]. Human keratinocytes express various nAChR subunits and mAChR subtypes M_1_, M_3_, M_4_ and M_5_ [8,16,17]. They also actively participate in the cholinergic system of the skin, as they not only respond to Ach, but also synthesize and secrete it [14]. 

In addition to their canonical signaling pathways, mAChRs are often capable of activating further signaling pathways. As with many other GPCRs, mAChR signaling can indirectly activate the mitogen-activated protein (MAP) kinase cascade and therefore induce a phosphorylation of the extracellular signal regulated kinase (ERK) [18,19,20,21]. However, different signaling pathways are involved in the cholinergic ERK activation in different cell types. One key player in the cholinergic MAP kinase signaling cascade that has gathered increasing attention in recent years is the epidermal growth factor receptor (EGFR). EGFR belongs to the family of receptor tyrosine kinases and activates various signaling pathways, including MAP kinases, upon growth factor stimulation. However, numerous studies have shown that EGFR can also be activated by means of a transactivation cascade after stimulation of various GPCRs [22,23]. Predominantly, EGFR transactivation is mediated by the so-called “triple-membrane-passing signal” (TMP) that involves the activation of matrix metalloproteinases (MMPs), leading to a proteolytic release of ligands of the EGF family which then transmit the signal by activating the EGF/ERB-B receptor family members [24,25]. Transactivation of EGFR provides an opportunity for additional intracellular signaling pathways to transduce GPCR signals and this crosstalk gives rise to a complex signaling network. Many GPCRs and other receptors are capable of activating the MAP kinase pathways through various signaling routes, including the EGFR transactivation mode known to be important for the mAChR signaling [22,23,26,27,28,29,30,31,32,33,34].

Even though it has become clear that ACh plays an important role in the human skin and keratinocytes, little is known about the details of cholinergic signal transduction and its interplay with other signal transduction pathways in these cells. The aim of our study was to characterize the molecular details of cholinergic activation of the MAP kinase pathways in HaCaT cells and to dissect the possible role of EGFR transactivation in this process. HaCaT cells represent a good model for human keratinocytes [35], and they are widely used as an immortal alternative for primary skin keratinocytes as they exhibit normal keratinization and can even be used for organotypic skin cultures [35,36]. HaCaT cells have been shown to express at least the M_3_ receptor subtype and to respond to stimulation with ACh [37,38]. They also express EGFR family members (EGFR, Erb-B2 and Erb-B3) and react to stimulation with EGFR ligands [39]. Furthermore, ligands of the EGF family have been shown to be important regulators of HaCaT proliferation and differentiation [36].

We here show that activation of the MAP kinase ERK and Akt (also known as protein kinase B) by a cholinergic stimulus in HaCaT cells requires EGFR transactivation. MAP kinase activation takes place by means of the MMP dependent TMP pathway. On the other hand, Akt activation also involves phosphatidylinositol 3-kinase (PI3K) and may involve Src kinase activity, which are not required for ERK activation. Furthermore, we show that cholinergic EGFR activation does not result in a considerable degree of receptor internalization, and the phosphorylation pattern and EGFR ubiquitination also differ from direct EGF stimulation. These data suggest that ligands other than EGF, TGF-α or HB-EGF are likely to be involved in cholinergic EGFR transactivation in HaCaT cells. 

## 2. Results and Discussion

### 2.1. Results

Activation of the ERK kinases upon stimulation of the mAChRs has been shown to involve transactivation of the EGFR. However, also other signaling mechanisms can lead to ERK activation upon mAChR stimulation, and the degree of EGFR involvement varies considerably, depending, for example, on the cell type. We have here characterized the role of EGFR in ERK activation by cholinergic stimuli in HaCaT keratinocytes. In addition, we analyzed the role of EGFR in the activation of Akt, a kinase that is important for cell survival. 

**Figure 1:**
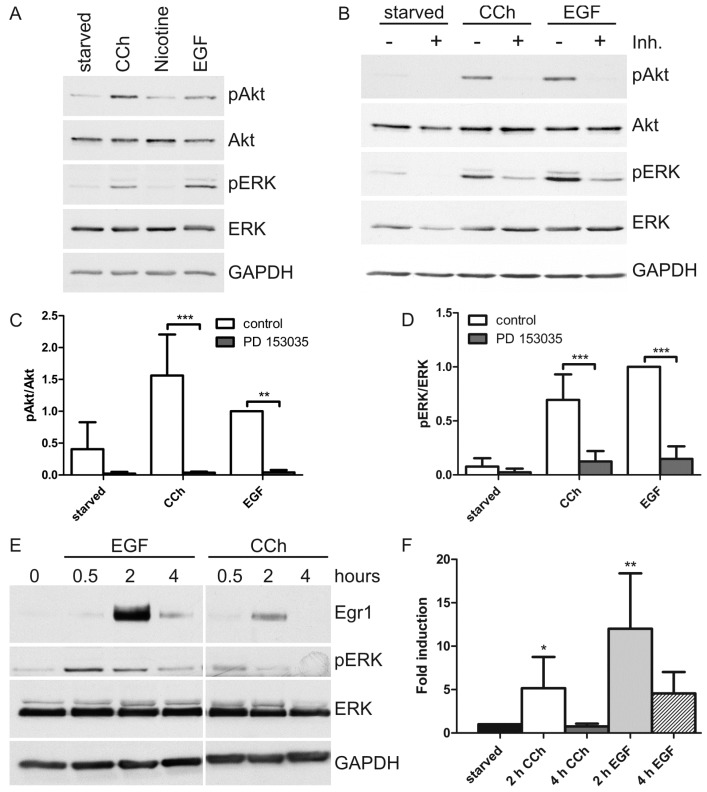

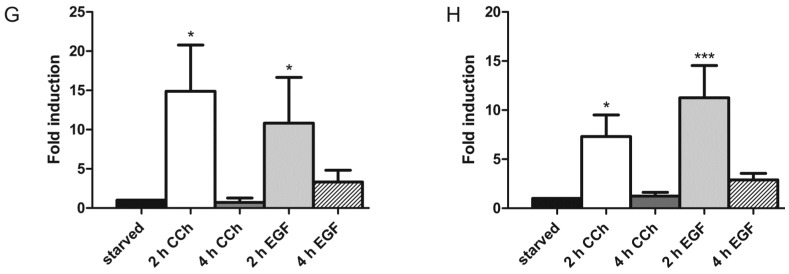
Inhibition of epidermal growth factor receptor (EGFR) kinase activity prevents cholinergic activation of Akt and extracellular signal regulated kinase (ERK). (**A**) Serum-starved HaCaT cells were stimulated with 1 mM CCh, 100 µM nicotine or 16 nM EGF for 30 min. Equal amounts of cell lysates were separated by SDS-PAGE and immunoblotted. The results are representative of three independent experiments; (**B**) To inhibit EGFR kinase activity, starved HaCaT cells were pretreated with the EGFR kinase inhibitor PD 153035 or a non-inhibiting analogue AG9 (both 1 µM) for 5 min. Control cells here represent the AG9 treated cells. The cells were then stimulated with 1 mM CCh or 16 nM EGF for 30 min in the presence of the inhibitor or its analogue. Equal amounts of cell lysates were separated by SDS-PAGE and immunoblotted; (**C**,**D**) The amount of pAkt and pERK was determined by densitometric quantification and normalized to total Akt or ERK. Data are shown relative to the EGF stimulated control. Bars represent the mean ± SD of four independent experiments. Statistical analysis was performed with two-way ANOVA; ** *p* < 0.01, *** *p* < 0.001; (**E**–**H**) Transcriptional response upon EGF or CCh stimulation (30 min–4 h) was measured at protein (**E**, Egr1 expression) or mRNA level (measured with quantitative real-time PCR) for Egr1 (**F**), cFos (**G**), and Dusp1 (**H**). Bars represent the mean ± SD of three independent experiments. Statistical analysis was performed with one-way ANOVA; * *p* < 0.05, ** *p* < 0.01, *** *p* < 0.001. (**E**) Shows a representative result of three independent experiments.

To study if a cholinergic stimulus leads to activation of ERK and Akt, HaCaT cells were stimulated for 30 min with the cholinergic agonist carbachol (CCh, 1 mM), EGF (16 nM) or nicotine (100 µM), and the activation of Akt and ERK was analyzed with phospho-specific antibodies (Figure 1A). Whereas stimulation with CCh and EGF resulted in a robust activation of both Akt and ERK, nicotine did not induce any activation of ERK and only a minimal change in Akt phosphorylation. Thus, the activation of ERK and Akt in HaCaT cells by cholinergic stimuli is likely to be mediated by the muscarinic receptors.

The effect of EGFR inhibition on the activation of ERK and Akt upon 30 min CCh stimulation was next tested. As a control, direct EGF stimulation was used. HaCaT cells were starved for 24 h and then stimulated with CCh or EGF for 30 min, and the activation of ERK and Akt was again measured (Figure 1B). A strong activation of ERK was observed with both CCh and EGF, whereas Akt activation was significantly increased only in the case of CCh, but not so upon EGF stimulation (Figure 1C). This was due to the high but variable basal activity of Akt observed in starved cells, which prevents the results from becoming statistically significant, although a clear trend towards activation by CCh and EGF was seen in all experiments shown here and later. The activity of Akt (including the basal one) was fully and highly significantly suppressed by the EGFR kinase inhibitor PD 153035 (Figure 1C), and this inhibitor also significantly reduced ERK activation to the level of basal ERK phosphorylation observed in starved cells (Figure 1D). Thus, the activation of both Akt and ERK upon CCh stimulation in HaCaT cells is basically dependent on EGFR activation, although a quantitative analysis of Akt activation is complicated by the high basal level of activity. Activation of ERK usually culminates into a transcriptional response during which early genes such as Egr1 (early growth response 1) are activated, which then in turn enhance the transcription of further genes such as cFos and the dual specificity phosphatase Dusp1. This was measured using quantitative real-time PCR (RT-qPCR). Stimulation of HaCaT cells for 2 h with either CCh or EGF resulted in increased protein expression of Egr1 (Figure 1E) and increased amount of the mRNA for Egr1 (Figure 1F), cFos (Figure 1G) and Dusp1 (Figure 1H), implicating that CCh also induces a growth response in HaCaT cells.

**Figure 2:**
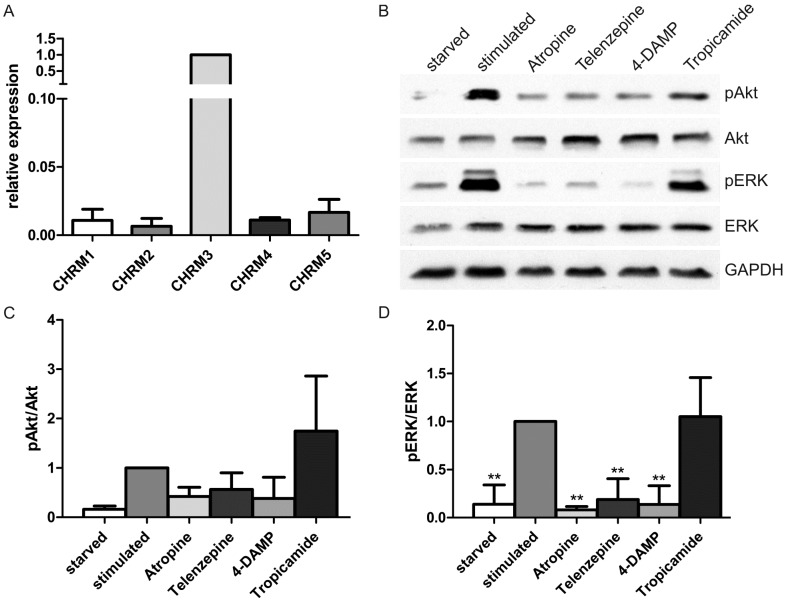
Inhibition of M_1_ and M_3_ receptors prevents cholinergic activation of ERK. (**A**) The expression of the muscarinic receptor subtypes on mRNA level in HaCaT cells was studied using RT-qPCR; (**B**) Serum-starved HaCaT cells were pretreated with mAChR inhibitors for 30 min and then stimulated with 1 mM CCh for 30 min in the presence of the inhibitors. M_1_ was inhibited with 10 µM telenzepine, M_3_ with 10 µM 4-DAMP and M_4_ with 10 µM tropicamide. As a control, 25 µM atropine was used to inhibit all muscarinic receptors. Equal amounts of cell lysates were separated by SDS-PAGE and immunoblotted; (**C**,**D**) The amount of pAkt and pERK was determined by densitometric quantification and normalized to total Akt or ERK. Data are shown relative to CCh stimulated cells. Bars represent the mean ± SD of three independent experiments. Statistical analysis was performed with one-way ANOVA; ** *p* < 0.01.

Since our data pointed to involvement of the mAChRs, their expression was tested in HaCaT cells by RT-qPCR. Analysis of the relative mRNA amounts demonstrated that the mRNA for CHRM3 (corresponding to M_3_) was the most abundant one, whereas all other mAChRs were only expressed at a lower level (Figure 2A). To characterize which mAChRs are responsible for the activation of ERK and Akt, various mAChR inhibitors were used. The general mAChR antagonist atropine resulted in reduced Akt activity and blocked ERK activation to the level observed in unstimulated cells (Figure 2B–D). Telenzepine, which inhibits mainly M_1_, and 4-DAMP, an inhibitor of M_3_, resulted in a highly significant ERK inhibition, whereas tropicamide, a M_4_ specific inhibitor, did not show any inhibitory effect on ERK activation (Figure 2D). Similar data were obtained in the case of Akt activation (Figure 2C), but the inhibition was less profound than that of ERK and not statistically significant. Thus, these data show that M_3_ and M_1_ are involved in CCh mediated activation of at least ERK, consistent with the expression of these receptor types in HaCaT cells. As none of the inhibitors resulted in significant inhibition of Akt activity, it is not possible to state which receptor type is involved in Akt activation.

**Figure 3:**
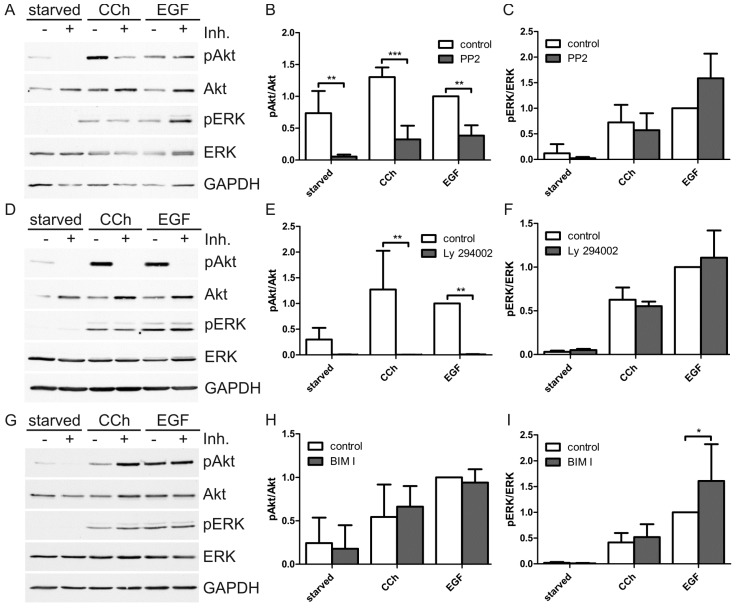
Src family kinases and PI3K are involved in cholinergic Akt but not ERK activation. (**A**,**D**,**G**) Serum-starved HaCaT cells were pretreated with the Src family specific inhibitor PP2 and the non-inhibiting PP3 (both 10 µM) for 10 min (**A**), or with 20 µM PI3K inhibitor Ly 294002 or DMSO for 10 min (**D**), or with 200 nM BIM I, a PKC inhibitor, or DMSO for 20 min (**G**). The cells were then stimulated with 1 mM CCh or 16 nM EGF for 30 min in the presence of the inhibitors. Equal amounts of cell lysates were separated by SDS-PAGE and immunoblotted; (**B**,**C**,**E**,**F**,**H**,**I**) The amount of pAkt and pERK was determined by densitometric quantification and normalized to total Akt or ERK. Data are shown relative to the EGF stimulated controls. Bars represent the mean ± SD of at least three independent experiments. Statistical analysis was performed with two-way ANOVA; * *p* < 0.05, ** *p* < 0.01, *** *p* < 0.001.

Activation of ERK by cholinergic signals has been shown to involve intracellular signaling by Src family kinases, protein kinase C (PKC) or PI3K [40,41,42], depending on the cell type analyzed. Specific inhibitors of these kinases were used to study if they are involved in cholinergic ERK and Akt signaling in HaCaT cells (Figure 3). Src kinases were inhibited with PP2 (Figure 3A–C), PI3K with Ly 294002 (Figure 3D–F) and PKC with BIM I (Figure 3G–I). Interestingly, although both PP2 and Ly 294002 significantly inhibited Akt activation (including basal activity and activation by CCh and EGF; Figure 3B,H), they showed no inhibitory effect on ERK activation either by CCh or EGF (Figure 3C,F). However, the PKC inhibitor affected neither Akt (basal or stimulated) nor ERK activity (Figure 3H,I). In fact, ERK activation by EGF appeared even to be stimulated upon PKC inhibition (Figure 3I). Thus, although both ERK and Akt activation by CCh are dependent on EGFR transactivation (Figure 1B–D), the downstream signaling pathways from EGFR towards ERK appear not to involve Src kinases and PI3K. On the contrary, even the basal activity of Akt appears to be dependent on both Src kinases and PI3K. 

**Figure 4:**
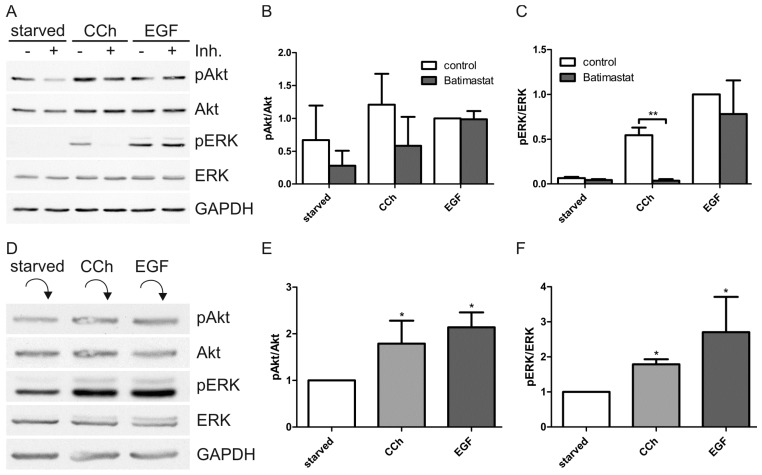
Cholinergic activation of ERK requires matrix metalloproteinases (MMPs) and extracellular ligand release. (**A**) Serum-starved HaCaT cells were pretreated with 10 µM broad spectrum MMP inhibitor batimastat or DMSO as a negative control and then stimulated with 1 mM CCh or 16 nM EGF for 30 min in the presence of the inhibitor or DMSO. Equal amounts of cell lysates were separated by SDS-PAGE and immunoblotted for the indicated proteins; (**B**,**C**) The amount of pAkt and pERK was determined by densitometric quantification and normalized to total Akt or ERK. Bars represent the mean ± SD of three independent experiments. Statistical analysis was performed with two-way ANOVA; ** *p* < 0.05; (**D**) Serum-starved HaCaT cells were stimulated with 1 mM CCh or 16 nM EGF for 15 min, after which fresh medium was added to allow for ligand release (15 min). The medium was collected and given to starved cells which were collected after 15 min. Equal amounts of cell lysates were separated by SDS-PAGE and immunoblotted; (**E**,**F**) The relative amount of pAkt and pERK was quantified as in (**B**,**C**). Bars represent the mean ± SD of four independent experiments. Statistical analysis was performed with Student’s *t*-test; * *p* < 0.05.

MMPs have been shown to be activated by mAChRs upon cholinergic stimuli, resulting in release of EGF-like ligands from the cell surface, which then can activate EGFR [27,28,34]. Inhibition of MMPs with the broad range MMP inhibitor Batimastat resulted in almost complete inhibition of ERK activation upon CCh (Figure 4A,C) and a non-significant reduction of Akt activation (Figure 4B), without any significant effect on direct EGF induced ERK and Akt activation. This implicates that MMPs are involved in the transactivation of EGFR upon cholinergic stimulus in HaCaT cells. To study this further, HaCaT cells were stimulated with CCh or EGF, thoroughly washed to remove any surface bound ligand, and incubated with fresh serum-free medium to allow the release of EGF-like ligands from these cells. The medium was then collected and given to unstimulated cells which were analyzed after 15 min incubation for ERK and Akt activation. Both Akt and ERK were significantly activated by conditioned medium from CCh and EGF stimulated cells as compared to medium from starved cells (Figure 4D–F).

**Figure 5:**
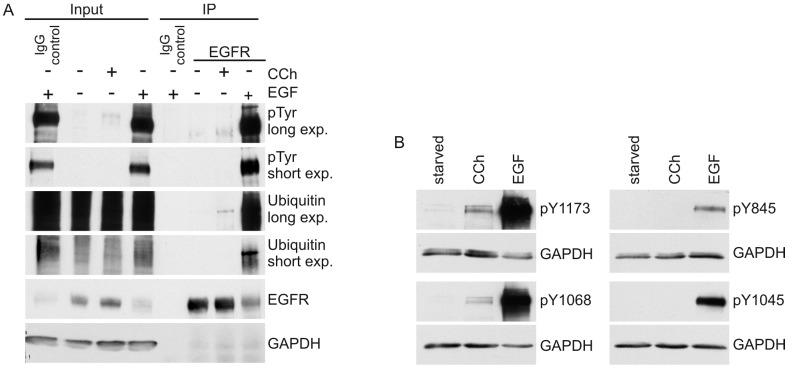
CCh treatment leads to a lower degree of phosphorylation and ubiquitination of the EGFR than EGF treatment. Serum-starved HaCaT cells were stimulated with 1 mM CCh or 16 nM EGF for 15 min (**A**) or 30 min (**B**). (**A**) The cells were lysed and EGFR was immunoprecipitated. The samples were separated by SDS-PAGE and immunoblotted for phospho-Tyr, ubiquitin and EGFR; (**B**) Equal amounts of cell lysates were separated by SDS-PAGE, immunoblotted and probed with anti phospho-Tyr antibodies specific for the indicated EGFR phosphorylation sites. The blots shown are representative of three independent experiments.

Upon stimulation with EGF, EGFR becomes phosphorylated on several Tyr residues and is ubiquitinated. These modifications affect both the downstream signaling response of the receptor and its cellular trafficking, including endocytosis and degradation (reviewed in [43]). We thus tested if CCh stimulation also results in Tyr phosphorylation (Tyr-P) and ubiquitination of EGFR which was immunoprecipitated from EGF and CCh stimulated cells. As expected, EGF resulted in a robust Tyr-P and ubiquitination of EGFR (Figure 5A), whereas CCh produced a merely detectable signal for Tyr-P or ubiquitination. Although the signal for Tyr-P was altogether weak after CCh stimulation, we next tested the phosphorylation of specific Tyr residues in EGFR (Figure 5B). Tyr-1173 and -1068 become autophosphorylated and direct the downstream signal towards MAP kinase activation, whereas Tyr-1045 is a binding site for the ubiquitin ligase c-Cbl which mediates EGFR ubiquitination [44]. Tyr-845 in turn becomes phosphorylated by Src kinases [45]. Phosphorylation of Tyr-1173 and Tyr-1068 was detected after CCh stimulation, although it was much weaker than EGF induced Tyr-P (Figure 5B). Consistent with the low degree of ubiquitination, CCh did not induce phosphorylation of Tyr-845, nor was any Tyr-P of Tyr-1045 observed.

**Figure 6:**
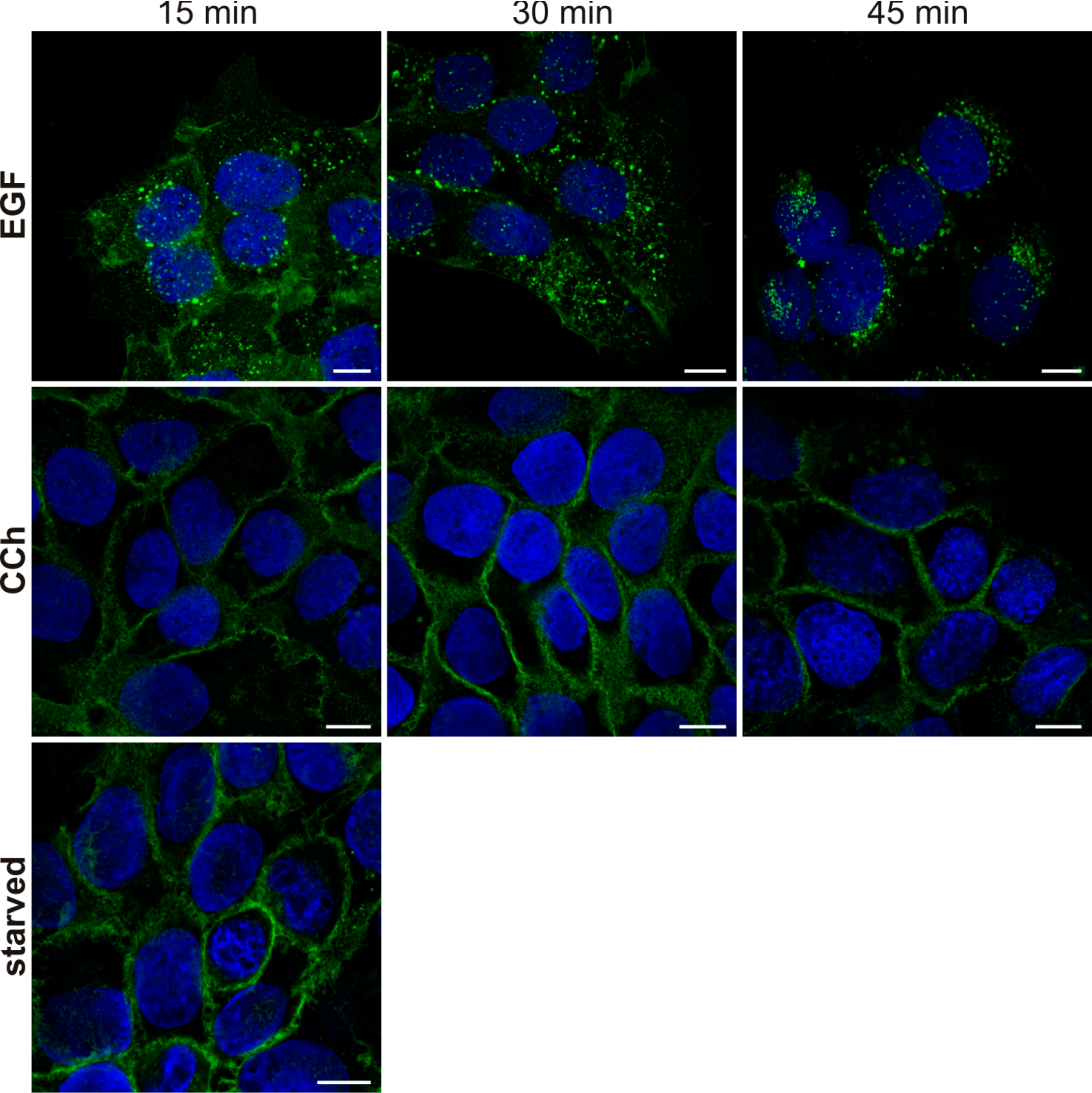
EGFR is internalized after EGF but not after CCh treatment. Serum-starved HaCaT cells were stimulated with 1 mM CCh or 16 nM EGF for 15, 30 or 45 min. Cells were fixed with methanol and immunostained for EGFR. Nuclei are shown in blue (DAPI staining). The results are representative of three independent experiments. Scale bar: 10 µm.

**Figure 7:**
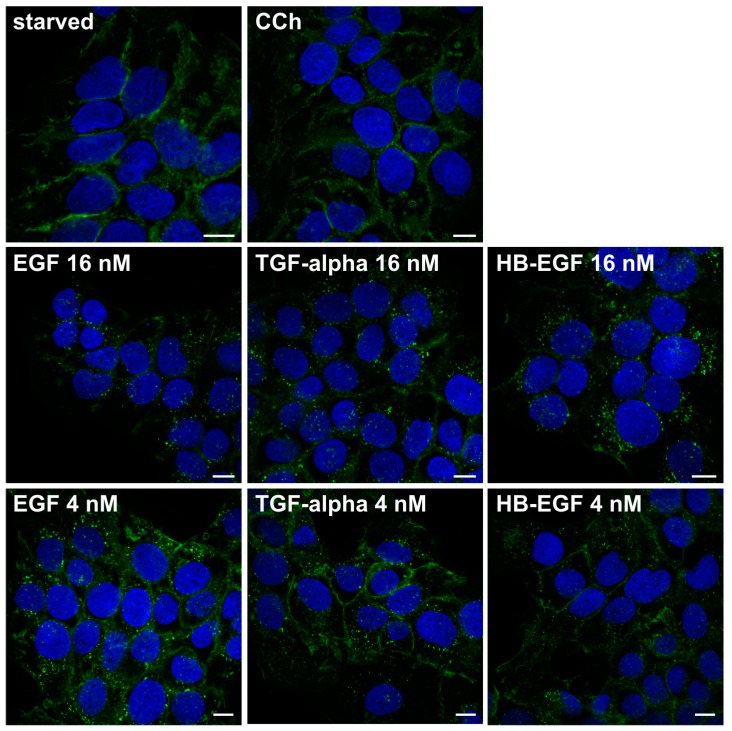
Cholinergic stimulation does not induce EGFR endocytosis, whereas EGF, TGF-α and HB-EGF do. EGFR localization after CCh, EGF, TGF-α and HB-EGF treatment was studied using immunofluorescence staining. Serum-starved HaCaT cells were stimulated with 1 mM CCh, 16 or 4 nM EGF, TGF-α or HB-EGF for 30 min. Cells were fixed with methanol and immunostained for EGFR. Nuclei are shown in blue (DAPI staining). The results are representative of three independent experiments. Scale bar: 10 µm.

EGF stimulation results in endocytosis of the EGFR from the plasma membrane and in degradation in lysosomes. When HaCaT cells were stimulated with EGF for 15–45 min (Figure 6), most of the EGFR became localized in intracellular vesicles which have previously been shown to be late endosomes/lysosomes [46]. However, CCh was unable to induce any detectable degree of endocytosis even after prolonged stimulation for 45 min. Thus, these findings suggest that the transactivation of EGFR in HaCaT cells may not be mediated by EGF, but by another ligand of the EGF-like growth factor family. This would also be consistent with the fact that although EGF is important for epidermal physiology *in vivo*, it is not produced by the keratinocytes but by the fibroblasts [39]. Heparin binding EGF (HB-EGF) and transforming growth factor α (TGF-α) are typical ligands that bind to EGFR and have been shown to be involved in EGFR transactivation by other GPCRs [47,48]. We thus tested if these ligands might be involved in cholinergic EGFR transactivation. However, both TGF-α and HB-EGF (16 and 4 nM) robustly induced EGFR endocytosis in HaCaT cells (Figure 7), implicating that they are unlikely to be responsible for the EGFR activation upon cholinergic stimulation, although their role in this process cannot be completely excluded. 

### 2.2. Discussion

**Figure 8:**
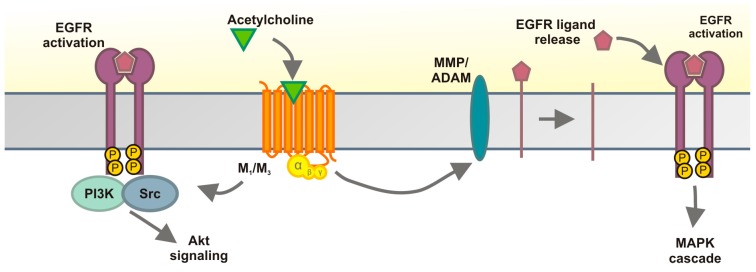
Cholinergic EGFR transactivation in HaCaT cells. Stimulation of mAChRs results in MMP activation and release of an EGF family ligand that activates the EGFR. Downstream thereof, MAP kinase ERK and the Akt kinase are activated, but ERK and Akt activation proceed through different pathways, since Src kinases and PI3K are required for Akt activation but are dispensable for ERK activation by cholinergic signaling. See Discussion for details.

Our above data show that stimulation of muscarinic but not nicotinic acetylcholine receptors in HaCaT cells results in activation of EGFR and its downstream signaling cascades towards MAP kinase and Akt (Figure 8). Both ERK and Akt activation by cholinergic stimulus are strictly dependent on EGFR kinase activity in these cells, and the MMP mediated TMP signaling route plays an important role in EGFR and MAP kinase activation. Using receptor subtype specific inhibitors, we could show that M_3_ and M_1_ are involved in cholinergic EGFR and MAP kinase activation in HaCaT cells. This is consistent with our data that the mRNAs for all five mAChRs are present in HaCaT cells, although the mRNA for M_3_ is clearly the most abundant one. Our expression data are different from those presented by Metzger *et al.* [37] who were only able to detect M_3_ in these cells. However, this could be due to the fact that the said study used a semi-quantitative PCR assay which is far less sensitive than our RT-qPCR approach. However, due to a lack of specific antibodies for the mAChRs that would facilitate the detection of endogenous mAChRs, which may be expressed only in low levels in HaCaT cells, it was not possible to show the receptor expression by means of Western blot. Nevertheless, our inhibitor data strongly suggest that in addition to the abundant M_3_, also M_1_ is expressed and plays a role in EGFR transactivation. The individual contributions of these receptors would need to be analyzed by means of knockdown experiments in future studies.

To our knowledge, the cholinergic activation of MAP kinase and Akt signaling has not been studied in detail in HaCaT cells. Cholinergic MAP kinase activation has been studied in various cell lines, but most studies addressing EGFR transactivation by cholinergic signaling or by other GPCRs have neglected to analyze the activation of Akt (for examples see [22,23,31,49,50,51]). Here, we could clearly show that cholinergic ERK activation is fully dependent on EGFR activation and MMP activity. However, the analysis of Akt activation by cholinergic stimuli in our study was generally complicated by the high basal activity of Akt observed already in starved cells. Thus, the effect on Akt activity of CCh stimulation as compared to starved cells in some experiments remained relatively vague. Dissection of cholinergic Akt activation in cells that express only endogenous mAChRs appears to be generally difficult, as this has been addressed only by very few studies. McCole *et al.* [49] could show in colon epithelial cells that the inhibitory effect observed on CCh stimulated chloride secretion is due to EGFR transactivation and requires PI3K/Akt activity. However, similar to our study, CCh stimulation induced only an insignificant Akt activation; although a clear tendency towards CCh mediated Akt activation could be observed. Akt was significantly activated only when phosphatase inhibitors were used during stimulation, implicating that phosphatases actively prevent Akt activation upon CCH stimulus [49]. This could also explain the high variability in Akt activity observed in our study. Nevertheless, we would like to stress that although the Akt activation data in our study needs to be interpreted with some reservation due to lack of significance of CCh stimulation in some experiments, the Src and PI3K inhibitors showed a clear and statistically significant reduction of Akt activity. As these inhibitors also clearly reduced the basal Akt activity, our data implicate that these kinases are already involved in the basal activation of Akt in starved cells.

An interesting outcome of our study was that although ERK and Akt activation upon cholinergic stimulus both require EGFR activity, only ERK activation is fully dependent on the MMP mediated extracellular pathway of EGFR activation, whereas MMP inhibition only partially inhibits the basal and stimulated Akt activity (Figure 8). Interestingly, it appears that an intracellular signaling route involving Src kinases and PI3K is at least in part required for Akt activity, whereas inhibitors of these two kinases show no effect on ERK activation upon cholinergic stimulation. Akt activation could thus be mediated by direct phosphorylation of EGFR by Src kinases, whereas autophosphorylation of other residues after ligand stimulation might be required for ERK activation. However, we were not able to detect any phosphorylation of Tyr845, a Src phosphorylation site in EGFR [45], after cholinergic transactivation, implicating that at least this residue is not directly phosphorylated by Src kinases during transactivation. Thus, Src kinases may phosphorylate other residues in EGFR or other proteins that then mediate EGFR activation. This appears to be at least partially ligand independent, since MMP activity and thus ligand release are not essential for Akt activity (basal or stimulated) in HaCaT cells. 

In general, the magnitude of EGFR phosphorylation upon cholinergic stimulation was much lower than that induced by EGF directly. Nevertheless, we observed a clear phosphorylation of Tyr1173 and Tyr1068 of the EGFR upon CCh, whereas Tyr1045 and Tyr845 were not phosphorylated. These data are consistent with those of McCole *et al.* [49] who could show in intestinal epithelial cells that CCh induces a lower degree of phosphorylation of the EGFR than direct stimulation with EGF and TGF-α. Both Tyr1173 and Tyr1068 are major autophosphorylation sites of the EGFR which mediate the binding of the adaptor protein Growth factor receptor-bound protein 2 (Grb2) which is important for the activation of the MAP kinase signaling downstream of the EGFR [52,53]. We here observed both a reduced Tyr phosphorylation of the EGFR and a much lower degree of ubiquitination upon cholinergic transactivation as compared to direct EGF stimulation. This is also consistent with the low degree of endocytosis observed upon cholinergic stimulation (see below), as ubiquitination of EGFR is associated with EGFR degradation in lysosomes [43,54,55].

Of the eight EGF family ligands, HB-EGF and TGF-α have been shown to be the main ligands that mediate EGFR transactivation upon GPCR stimulation [48], but also amphiregulin (AREG) appears to be involved in some cases [47,56]. Epidermal keratinocytes have been shown to express not only HB-EGF and TGF-α [57,58], but also AREG and epiregulin (EREG) [59,60], which are also expressed in HaCaT cells (our unpublished data). Furthermore, TGF-α has been shown to be important for tissue regeneration in organotypic cultures of HaCaT cells [36], demonstrating that EGF family ligands play an important role in the skin physiology (reviewed in [61]). At present, we do not know which ligand is responsible for the cholinergic EGFR activation in HaCaT cells. However, the fact that we could not observe any considerable endocytosis in our immunofluorescence experiments suggests that a ligand not capable of inducing EGFR endocytosis is released upon CCh stimulation. Alternatively or even additionally, a thus far unidentified intracellular signaling route that is activated by the mAChRs might prevent EGFR endocytosis despite the release of ligands that normally stimulate receptor endocytosis. One potential candidate for such modifier is calcium which is frequently involved in mAChR signaling.

Interestingly, the downstream signaling events and EGFR trafficking have been shown to be different for the individual EGF family ligands [46]. Although EGF, HB-EGF, TGF-α, AREG, EREG and betacellulin all can induce an uptake of EGFR from the plasma membrane, a very high concentration of some of them is required. Furthermore, the uptake kinetics and the post-endocytic sorting are considerably different among these ligands, with HB-EGF and betacellulin being most efficient inducers of endocytosis and degradation [46]. This makes a role for HB-EGF in EGFR transactivation in HaCaT cells unlikely, as no endocytosis of the EGFR could be observed even when low nanomolar concentration of HB-EGF was used. Interestingly, EREG only induces a considerable degree of endocytosis when concentrations in a higher nanomolar range are used [46]. A maximal internalization of the EGFR by the different ligands is observed beyond 10 min, and recycling of the EGFR becomes evident when longer time points are used [46]. Therefore, we performed time-scale experiments (15–45 min) for the endocytosis, where the shortest time point used should have revealed any endocytosis of the EGFR as internal staining. This was indeed observed for the EGF family ligands but not for CCh in our experiments. Thus, it appears that the ligand released upon CCh stimulation is not an efficient endocytosis inducer. Furthermore, it is likely that only low concentrations of the ligand are released by cholinergic stimuli. However, EGF concentrations as low as 0.8 nM are capable of inducing EGFR endocytosis in HaCaT cells (our unpublished data), again suggesting an involvement of another ligand. In future experiments, we are aiming at characterizing the expression profiles of EGF family ligands in HaCaT cells and in identifying the ligand released by cholinergic stimuli.

Cholinergic EGFR transactivation has so far been studied in various cell types. In many cases, overexpression systems in e.g. human embryonic kidney (HEK) or African green monkey (COS7) cells have been used to study receptor transactivation [23,40,41,42]. Due to this, heterogeneous data about the involvement of various signaling proteins such as Src kinases and PKC in EGFR transactivation have been obtained. This stresses the importance of analyzing the molecular details of EGFR transactivation and its physiological significance in the cell line of interest, and shows that literature findings from another cell type cannot be directly adapted to a further cell line. We have here provided a detailed analysis of the cholinergic transactivation of EGFR and its downstream signaling cascades in HaCaT keratinocytes. By means of EGFR/MAP kinase transactivation, cholinergic stimulation is important for the regulation of cell proliferation, which may have implications in cancer. It has also been shown to regulate cell migration, and Metzger *et al.* [37] could show that CCh displays an inhibitory effect on HaCaT cell migration induced by EGF. Some mAChRs can also inhibit the migration of primary human keratinocytes [62]. Thus, it will be of importance to dissect the involvement of the individual signaling components that affect a certain physiological process such as proliferation, ion currents, secretion and migration, which are all under cholinergic control in many cell types. This way, we will gain molecular insight into the regulation of the evidently complex process of EGFR transactivation by cholinergic signals. Characterization of the crosstalk between cholinergic and EGFR signaling is also especially important considering the importance of both the EGF family ligands and ACh in the homeostasis of the epidermis.

## 3. Experimental Section

### 3.1. Reagents

Human EGF, CCh, nicotine, atropine, telenzepine, 4-DAMP, tropicamide, PP2 and Ly 294002 were purchased from Sigma-Aldrich (Taufkirchen, Germany). PD 153035, AG9, batimastat, PP3 and Bisindolylmaleimide I (BIM I) were obtained from Calbiochem (Merck Millipore, Darmstadt, Germany). Human TGF-alpha and human HB-EGF were purchased from PeproTech (Hamburg, Germany).

### 3.2. Cell Culture

HaCaT cells (obtained from Dr. Boukamp, German Cancer Research Center, Heidelberg, Germany) were cultured in Dulbecco’s Modified Eagle’s Medium (DMEM, Life Technologies, Carlsbad, CA, USA) high glucose, supplemented with 10% fetal calf serum, 1% penicillin/streptomycin, 1% sodium pyruvate and 1% non-essential amino acids (all from Life Technologies). The cells were grown in a humidified incubator at 37 °C and 8% CO_2_.

### 3.3. Cell Stimulation and Inhibitor Treatment

HaCaT cells were grown to 80%–100% confluency and starved in serum-free medium for 24 h. Cells were stimulated with nicotine (100 µM), CCh (1 mM) or EGF (16 nM) for 30 min, or as indicated in qPCR experiments, in serum-free medium. Control cells were treated with serum-free medium without any stimulant. In some experiments, the cells were pretreated with pharmacological inhibitors for different times and concentrations. Telenzepine, 4-DAMP and tropicamide were used at 10 µM and atropine at 25 µM concentration and added 30 min before the stimulation. PD 153035 and AG9 were preincubated for 5 min with a concentration of 1 µM. PP2 and PP3 were used at 10 µM and added 10 min before stimulation. BIM I preincubation (200 nM) was performed for 20 min, whereas Ly 294002 (10 µM) was added to the cells 10 min prior to stimulation. Batimastat (10 µM) was preincubated for 30 min.

### 3.4. Antibodies

Monoclonal mouse antibodies against pAkt (Ser473) and pEGFR (Tyr1068), polyclonal rabbit antibodies against Akt and pEGFR (Tyr1045 and Tyr845) and monoclonal rabbit antibodies against EGFR and pEGFR (Tyr1173) were purchased from Cell Signaling Technology (Danvers, MA, USA). Mouse monoclonal antibodies against pERK and ubiquitin and rabbit polyclonal antibodies against ERK and phosphotyrosine were from Santa Cruz Biotechnology (Santa Cruz, CA, USA). A mouse monoclonal antibody against GAPDH was obtained from Abcam (Cambridge, UK). Secondary antibodies goat anti-mouse and goat anti-rabbit coupled to horseradish peroxidase were obtained from Dako (Glostrup, Denmark).

### 3.5. Cell Lysis, Gel Electrophoresis and Western Blot

Cells were lysed in 50 mM Tris (pH 7.4), 150 mM NaCl, 2 mM EDTA and 1% Nonidet P-40 supplemented with protease inhibitor cocktail (Sigma-Aldrich), 1 mM sodium orthovanadate and 1 mM sodium fluoride. Protein concentration was measured with the protein assay reagent of Bio-Rad (Munich, Germany). Equal protein amounts were analyzed by SDS-PAGE and Western blot.

### 3.6. Ligand Release Assay

HaCaT cells were grown to 80%–100% confluency and starved in serum-free medium for 24 h. Cells were either left untreated or stimulated with CCh (1 mM) or EGF (16 nM) for 15 min. The medium was removed and the cells were washed with PBS. Fresh DMEM without serum was added and left on the cells for 15 min. Thereafter, the medium was transferred to fresh cells which had also been starved for 24 h. These cells were then incubated with the conditioned medium for 15 min.

### 3.7. Immunoprecipitation

Cells were lysed in immunoprecipitation buffer containing 10 mM Tris (pH 8.0), 150 mM NaCl, 5 mM EDTA, 0.5% Triton X-100 and 60 mM *n*-octyl-β-d-glucopyranoside supplemented with protease inhibitors, 1 mM sodium orthovanadate and 1 mM sodium fluoride. Mouse monoclonal anti-EGFR antibody (Santa Cruz) was used to precipitate EGFR, and a mouse monoclonal anti-myc antibody (Cell Signaling Technology) was used as an IgG control. The antibodies (2 µg) were precoupled to 20 µL of magnetic Dynabeads Protein G (Novex, Life Technologies). The cell lysates were incubated with the antibody-coupled Dynabeads for 16 h at 4 °C.

### 3.8. RNA Isolation and RT-qPCR

HaCaT cell total RNA was isolated using the Nucleospin^®^ RNA II isolation kit which includes a DNA elimination step (Macherey-Nagel, Düren, Germany) or peqGOLD TriFast™ (PEQLAB Biotechnologie GmbH, Erlangen, Germany), after which a separate DNAse digestion was performed. For qPCRs, 1–3 µg of RNA was reverse transcribed with oligo(dT) (25 µM) and random hexamer (30 µM) primers using the ProtoScript^®^II reverse transcriptase (for mAChRs) or M-MuLV reverse transcriptase (for other experiments; both from NEB, Frankfurt, Germany). RT-qPCRs (CFX Connect Real-Time PCR Detection System; Bio-Rad) were performed in triplicates with 0.8 µL of 5-fold diluted cDNA in 13 µL reaction mixtures using QPCR SensiMix™ SYBR (Bioline, Luckenwalde, Germany) or iTaq™ Universal SYBR Green Supermix (Bio-Rad, Munich, Germany). The annealing temperature was 60 °C for all PCR reactions. Primers were designed to be specific for the cDNA with PerlPrimer (Table 1). PCR products were quantified with the Δ*C*_t_-method. The geometric mean of the reference genes Rpl13a and GAPDH (for mAChRs) or Rpl13a, HPRT and Ywhaz (other experiments) was used for normalization.

**Table 1:** Primer sequences for RT-qPCR primers. All sequences are given in 5'–3' direction.

Gene	Primer Forward	Primer Reverse
*CHRM1*	CTGGTCAAGGAGAAGAAGGCGG	ACAGGGTCTCGGGAACACAGTC
*CHRM2*	CAAGGGAGAAAGAGAACCGGCA	ACCTGTCGCTGGTTTCGCTC
*CHRM3*	AGAAGAAAGCGGCCCAGACC	CACAGCCAGTAGCCCAGATTCC
*CHRM4*	TCACCAAGCCTCTCACCTACCC	TCCGCTTACCCACCACAAACTG
*CHRM5*	GAGAGGAAAGCAGCCCAGACAC	ACAACCAATAGCCCAAGTGCCA
*cFos*	TGGTGAAGACCGTGTCAGGAG	TGATCTGTCTCCGCTTGGAGTG
*Dusp1*	GGAGGACAACCAGGCAGAC	AGGTAAGCAAGGCAGATGGTGG
*Egr1*	TTCAACCCTCAGGCGGACAC	GTCTCCACCAGCACCTTCTCGT
*GAPDH*	CATCTTCCAGGAGCGAGATCCC	CCAGCCTTCTCCATGGTGGT
*HPRT*	GCAGTCCCAGGGTGCGTG	GGCCTCCCATCTCCTTCAT
*Ywhaz*	AGGTTGCCGCTGGTGATGAC	GGCCAGACCCAGTCTGATAGGA
*Rpl13a*	CCTGGAGGAGAAGAGGAAAGAGA	TTGAGGACCTCTGTGTATTTGTCAA

### 3.9. Immunofluorescence

HaCaT cells were grown on coverslips, starved in serum-free medium for 24 h and stimulated with CCh, EGF, TGF-α or HB-EGF as indicated. Cells were fixed with ice-cold methanol and labeled with mouse monoclonal anti-EGFR antibody (Santa Cruz) and anti-mouse Alexa Fluor 488-conjugated antibodies (Invitrogen, Life Technologies). Cells were mounted in Fluoromount (Sigma-Aldrich) supplemented with 50 mg/mL 1,4-diazadicyclo(2,2,2)octane (Fluka, Neu-Ulm, Germany). The specimens were analyzed using a Zeiss LSM710 Confocal Laser Scanning Microscope (Carl Zeiss, Jena, Germany).

### 3.10. Statistical Analysis

All experiments were performed at least three times. For the statistical analysis, Western blot bands of phosphorylated proteins were quantified by scanning densitometry using Quantity One software (Bio-Rad) and normalized to the total amount of the respective protein. Data are shown as the mean ± SD. For statistical comparison, one-way or two-way analysis of variance or Student’s *t* test were employed, as appropriate, using GraphPad Prism 4 (GraphPad Software, La Jolla, CA, USA). Values of *p* < 0.05 were considered significant (*), whereas values of *p* < 0.01 and *p* < 0.001 were defined very significant (**) and highly significant (***), respectively.

## 4. Conclusions

We have here shown that in human keratinocytes such as HaCaT cells, cholinergic stimulation of the mAChRs results in transactivation of the EGFR, which is required for the downstream activation of ERK and for Akt activity. Transactivation of EGFR in HaCaT cells induces a transcriptional response during which genes that regulate cell proliferation are activated. Thus, cholinergic signaling in the epidermis may be important for the regulation of cell proliferation and may even be associated with hyperproliferative diseases of the skin. In future experiments, it will be important to identify the EGF family ligands that are released upon cholinergic stimulus from HaCaT cells. Furthermore, although EGFR is evidently the main regulator of cholinergic signals towards MAP kinases in these cells, the fine tuning of the signaling, in terms of strength and duration, is regulated by the modifications (ubiquitination, phosphorylation, *etc*.) of the EGFR, its interaction partners, and by the dimerization partner of the EGFR. It will be important to characterize how the cholinergic EGFR signaling network differs from that observed upon direct activation, as intracellular factors activated by mAChRs are likely to modulate the signaling response.
